# A Study on the Mechanical Characteristics of Glass and Nylon Fiber Reinforced Peach Shell Lightweight Concrete

**DOI:** 10.3390/ma14164488

**Published:** 2021-08-10

**Authors:** Jawad Ahmad, Osama Zaid, Fahid Aslam, Muhammad Shahzaib, Rahat Ullah, Hisham Alabduljabbar, Khaled Mohamed Khedher

**Affiliations:** 1 Department of Civil Engineering, Military College of Engineering, NUST, Risalpur 24080, Pakistan; 2 Department of Civil Engineering, College of Engineering, Prince Sattam Bin Abdulaziz University, Al-Kharj 11942, Saudi Arabia; f.aslam@psau.edu.sa (F.A.); h.alabduljabbar@psau.edu.sa (H.A.); 3 Department of Civil Engineering, Harbin Institute of Technology, Harbin 150001, China; 20SF33383@stu.hit.edu.cn; 4 National Engineering Laboratory for High-Speed Railway Construction, School of Civil Engineering, Central South University, Changsha 410075, China; rahatullah@csu.edu.cn; 5 Department of Civil Engineering, College of Engineering, King Khalid University, Abha 61421, Saudi Arabia; kkhedher@kku.edu.sa; 6 Department of Civil Engineering, High Institute of Technological Studies, Mrezgua University Campus, Nabeul 8000, Tunisia

**Keywords:** peach shell aggregates, nylon fibers, glass fibers, lightweight concrete, strength

## Abstract

In the current study, the utilization of glass and nylon fibers in various percentages are added to enhance the mechanical performance of peach shell lightweight concrete. Glass and nylon fibers were added at 2%, 4%, 6%, and 8% by cement weight. The results showed that, as we added the glass and nylon fibers, the density of peach shell concrete was reduced by 6.6%, and the compressive, split tensile and flexural strength were enhanced by 10.20%, 60.1%, and 63.49%. The highest strength that was obtained in compressive, split tensile, and flexural strength at 56 days was 29.4 MPa, 5.2 MPa, and 6.3 MPa, respectively, with 6% of glass fiber in peach shell concrete. Mechanical test results showed that post-failure toughness and modulus of elasticity of peach shell concrete is enhanced with the utilization of fibers. To verify our lab results, a statistical analysis, such as response surface methodology, was performed to make a statistical model, it was confirmed by both lab results and statistical analysis that the mechanical performance of peach shell concrete could be significantly improved by adding glass fibers as compared to nylon fibers. With the use of fibers, the water absorption and porosity were slightly increased. Hence, the glass and nylon fibers can be used to improve the peach shell concrete mechanical properties to make concrete eco-friendly, sustainable, and lightweight.

## 1. Introduction

The utilization of modern agricultural or industrial waste materials to substitute the regular raw material in concrete has accomplished eco-friendly and sustainable improvement by decreasing raw material production expense [1,2]. The large use of crushed aggregates, for example, sand and crushed limestone to make concrete has led to severe issues of land pollution and cause huge problems for the environment [3,4,5]. Using lightweight aggregates, for example, shale, furnace slag, and pumice, rather than regular crushed limestone can make lightweight concrete [6,7]. Lightweight aggregate concrete has plenty of focal points, including better imperviousness to fire, heat protection, frost resistance, and sound absorption [8,9]. As of late, the usage of agricultural squanders, for example, coconut shell, peach shell, oil palm shell, and apricot shell, as a replacement for regular crushed aggregate is slowly growing. The advantages of utilizing agricultural waste rather than conventional aggregates to deliver lightweight aggregate concrete are to deal with waste materials and to decrease environmental issues [10,11,12]. Agricultural waste material is swiftly stowing in non-industrial nations, seed of peach organic product is called peach shell that causes eco-friendly behavior nearby their developed regions. More than a thousand tons of peach shell (PS) waste need discarding every year in China. The regenerative and lightweight trait of peach shells analyzed compared to the normal crushed aggregates could make peach shells one of the possible sustainable structure material and lightweight aggregate in the making of lightweight aggregate concrete. It might decrease land pollution brought about by peach shells and add to the reuse of peach shells. Enhancement in lightweight aggregate concrete mechanical properties relies upon the adding of binder materials, oven-dry density, particle size, water to cement ratio, and aggregate content. Past investigations indicated that lightweight aggregate concrete with more compressive strength could be made; however, the concrete was inclined to brittleness and poor elasticity because an aggregate that is lightweight is typically more brittle in strain (Elongation) [13,14]. The compressive strength capacity of lightweight aggregate concrete is 10 times more than its tensile strength [15,16]. Utilization of peach shells rather than normal crushed aggregates positively affects unit weight, and reduction of unit weight up to 25% might be accomplished. Flexural strength, modulus of elasticity, and split tensile strength of peach shell concrete are lesser as compared to lightweight aggregate concrete made of more lightweight aggregates, for example, extended shale and pumice, etc. Significant tensile cracking will occur in concrete under tension loading because of its lower tensile capacity [17]. Consequently, the improvement in peach shell concrete mechanical properties needs additional consideration [11,18].

Lightweight aggregate affects lightweight concrete properties [19]. Typically, the unit weight and strength may decrease with adding of lightweight aggregate. The bond between cement paste and lightweight aggregate is improved [20]. Matrix of lightweight concrete is ordered into air-cured foam concrete and autoclaved aerated concrete [21]. Autoclaved aerated concrete is a typical name for mortar of cement that contains gypsum and lime and an aerated agent is aluminum powder, and for improvement of strength, autoclaving is utilized. Air-cured cement mortar, which has entrained foam, is a typical name for cellular lightweight concrete. This concrete typically molds into block shape for simplicity of construction and transport, which is why it is called lightweight concrete block. It is helpful to utilize lightweight concrete with more strength, which is very important for structural members [22]. The requirement of structural lightweight concrete (SLC) has a base strength of 18.0 MPa and a unit weight of 1420–1890 kg/m^3^ (ACI 213R) [23]. Adding fibers is a compelling method to develop concrete mechanical properties, for example, flexural strength, split tensile strength, and other related characteristics [24]. The function of fibers in fiber-reinforced concrete is to improve the concrete performance through the effect of crack bridging and the interfacial bond of fiber binder aggregate [25,26]. Past examination demonstrated that fiber-reinforced concrete with two kinds of different fibers had resistance to crack development, impact resistance, and improved ductility. In the composite fiber material system, the fibers that are soft control the propagation of crack and also enhance ductility, while the fibers that are hard increase ultimate strength and also the first crack stress [27,28]. Yab et al. [29] declared that lightweight concrete with oil palm shells with 0.1% by volume of polypropylene (PP) and 0.9% by volume of steel fibers would be advised to improve in characteristics of flexural toughness [30].

Even though there are different studies on the inclusion of fibers in lightweight aggregate concrete, there is very limited research on the utilization of fibers in peach shell concrete. To make peach shell concrete like other lightweight aggregate concrete applied to reasonable structure components, for example, partition walls, parking garages, sidewalks, and roadblocks, the durability and mechanical properties of peach shell concrete must be additionally improved. The inclusion of fibers into peach shell concrete can make it a sustainable structure material and also make it ductile. Usually added fibers are nylon fiber, PP fiber, and steel fiber, and there are not many writings available on the utilization of glass fibers in lightweight aggregate concrete. The primary disadvantages of adding steel fibers to (LWAC) are decreased workability and a huge increase in unit weight. Polypropylene fiber is primarily utilized in concrete to improve impact resistance, ductility, and toughness, but do not expect to increase the strength [31]. Mastali et al. [32] revealed that the addition of glass fibers impelled a significant improvement in impact resistance, tensile and compressive strength of glass fiber-reinforced concrete. Compared to steel fibers, the benefit of adding nylon and glass fibers to peach shell concrete is that the unit weight is under 2000 kg/m^3^ and lesser as compared to a maximum recommended unit weight set for lightweight aggregate concrete. There are not many writings on the examination of nylon fiber-and glass fiber-reinforced lightweight peach shell concrete [4,33]

The reason for the current examination is to study the influence of two kinds of fibers (glass fiber and nylon fiber) and every fiber in different percentage of 2%, 4%, 6%, and 8% by weight on the mechanical performance of lightweight peach shell concrete. An examination of glass fiber-and nylon fiber-reinforced lightweight peach shell concrete is explored, the experimental tests on concrete include compressive, split tensile and flexural strength, density, workability, water absorption, residual compressive strength, porosity, and modulus of elasticity. Statistical analysis, i.e., response surface methodology (RSM) was adopted to make a statistical model to verify the research performed in an experimental trial to assists with growing information on the impact of glass fibers and nylon fibers with the different percentage to improve the mechanical properties of (PSFRC) peach shell fiber-reinforced lightweight concrete.

## 2. Material Properties

### 2.1. Cement and Silica Fume

For cement, 53-grade type I OPC [34] Portland cement is utilized acquired from a local market (Rawalpindi, Pakistan). To improve the cement performance, silica fume is used at 10% of cement weight in all mixtures. The physical properties and chemical composition of Portland cement are presented in Table 1.

### 2.2. Coarse and Fine Aggregates

**Figure 1:**
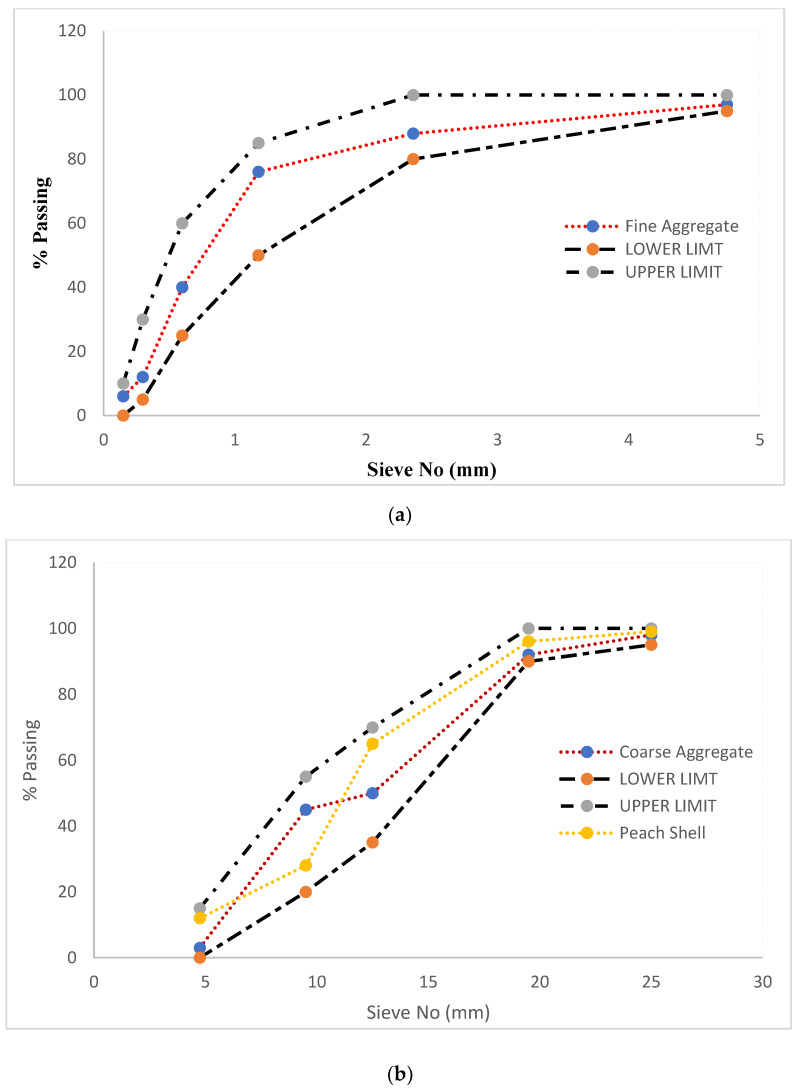
(**a**) Gradation curve of fine aggregate, (**b**) gradation curve of peach shell and coarse aggregate.

Crushed limestone is used as a normal-weight aggregate in the control sample and the peach shell is used as a replacement for crushed limestone. Unit weight of peach shells was 59% less as compared to typical coarse aggregate. Lightweight aggregates are not only good for reducing the dead load in buildings, but also have positive thermal properties (insulation). For fine aggregate, normal river sand was utilized and obtained from a nearby material supplier. Table 2 presents aggregate physical properties, while Figure 1 show the gradation curve of aggregates. Peach shells were obtained from a local supplier. Before adding, peach shells were washed to eliminate the residue and remaining dried peach mash from the peach shell surface. Using a crushing machine, a dried peach shell was squashed in the lab. To sieve the crushed peach shell, different sieves were used. Coarse aggregate lies in the range of size 4.75–20 mm. The crushed peach shell had a rough and irregular surface, and the peach shell aggregate had high porosity on the surface. High porosity on the outside of the peach shell reduces the unit weight and it paves the way that the peach shell has more water absorption capacity as compared to typical weight aggregate. Hence, the peach shell was lowered in water for 24 h and retained in surface dry conditions and an internal saturated dry condition before blending.

### 2.3. Water

Water for blending in cement had the pH value someplace in the scope of 6 and 8. In this study, clean drinking water was utilized, which was acquired from tube well property.

### 2.4. Superplasticizer

A high-range superplasticizer (SP) was utilized in the examination to increment the fresh concrete workability. At 1% of the cement weight, a superplasticizer is used in all blends.

### 2.5. Glass and Nylon Fibers

**Figure 2:**
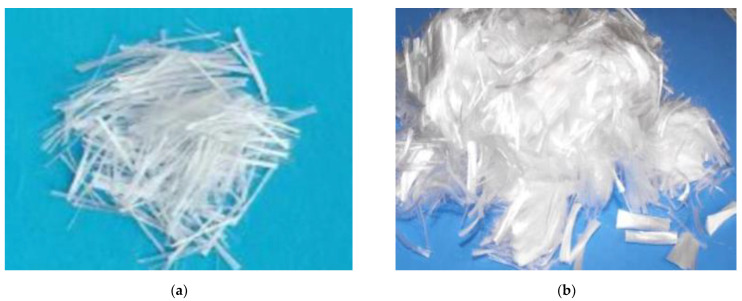
(**a**) Glass fiber and (**b**) nylon fiber.

Two different types of fibers are used in the present study, glass fiber and nylon fiber. The image and properties of glass fiber and nylon fiber are provided in Figure 2 and Table 3.

### 2.6. Mix Information

A sum of eight concrete blends was set up with various fiber proportions, and a control mix without the inclusion of fibers was additionally cast as a reference sample. The total sum of materials for all blends was equivalent, except for two different fibers (glass and nylon) and four fiber percentages. Fibers were added in different percentages like 2%, 4%, 6%, and 8% by weight, accordingly. Mix proportion of all concrete samples are provided in Table 4.

## 3. Sample Preparation and Testing Method

To make peach shell fiber reinforced concrete the following method was followed: initially, peach shell and sand from the river were filled into a mixture and dry blended for 3 min. Then, fibers and silica fume and cement were added to the blend and dry blended for 2 min. Fibers were manually distributed before they were blended. After that, 70% water blended in with a superplasticizer was incorporated into the blend and blended for 5 min. Following this, 30% water was added to the blend and blended for 7 min. After blending was over, then the test for the slump was performed right away, and afterward, the samples were cast in lubricated steel molds. Entire samples were compacted by a vibrator. Following compaction, all samples alongside the molds were enclosed by a sheet of plastic to forestall dampness. Samples were removed from the molds after 24 h. Lastly, samples were put away in a controlled environment with an overall moistness of 42% ± 3% and a temperature of 22 °C ± 3 °C till the experiment. The slump test was used to determine the workability of all blends as indicated by ASTM C143 [35]. The unit weight of entire samples was estimated as per ASTM C138 [36]. Then, 100 mm cube samples were utilized for the determination of compressive strength at the curing of 3, 7, 28, and 56 age. Split tensile strength and modulus of elasticity and entire blends were estimated at 28 days as indicated by ASTM C496 [37] and ASTM C469 [38]. The average estimation of three samples is taken for each test outcome. The water absorption test was done by the methodology recognized in ASTM C1585 [39]. Entire samples were oven-dried at 115 ± 2 °C for at least 24 h to eliminate dampness and, accomplish consistency before testing. The samples were at that point lowered in water for 24 h. The water absorption estimations of the samples were determined utilizing the accompanying equation.

(1) $$\mathrm{Water}\;\mathrm{absorption}\;(\%)=\frac{\left(M2-M1\right)}{M1}\;\times 100$$

In the above equation, *M*2 represents air surface-dried sample mass after drenching and *M*1 represents an air oven-dried sample mass. All samples’ porosity was determined as per ASTM C642 [40]. To determine the total porosity, absolute density is important by practice recognized in ASTM C642 [40]; subsequently, for the assertion of absolute density, a pycnometry technique is used. Values of open and total porosity of the sample may be determined by Equations (2) and (3) [41]. The volume of closed porosity might be acquired by deducting the volume of open porosity from total porosity volume.

(2) $$\mathrm{Total}\;\mathrm{Porosity}\;\mathrm{Volume}\;(\%)=\frac{\left(\rho a-\rho b\right)}{\rho a}\times 100.\;$$

Here, ρa is the sample absolute density (g/cm^3^) and ρb is the sample bulk density (g/cm^3^).

(3) $$\mathrm{Open}\;\mathrm{Porosity}\;\mathrm{Volume}\;(\%)=\frac{\left(M3-M1\right)}{\left(M3-M4\right)}\;\times 100$$

Here, *M*1 is the oven-dried sample mass; *M*2 is the surface-dried sample mass of the in-air after inundation; *M*3 is the surface-dried sample mass in air subsequently submersion and heating; and *M*4 is the sample apparent mass in water subsequently drenching and boiling.

## 4. Results and Discussion

### 4.1. Workability

**Figure 3:**
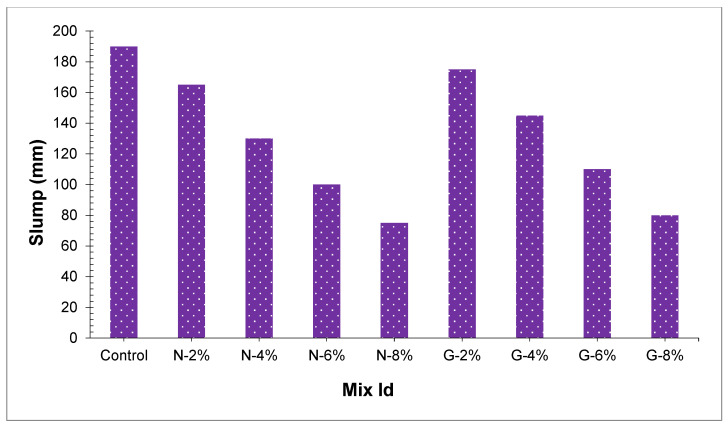
Slump values of fresh concrete.

The relationship between slump value and the fiber amount is presented in Figure 3. Yew et al. [15] studied, in oil palm shell concrete joining of various kinds of nylon fibers caused an impressive decline in slump value. The outcomes indicated that fresh PSC peach shell concrete slump values with nylon and glass fibers were reduced to 60 mm and 70 mm, individually. The reason for this occurrence might be clarified that the interfacial bond between fibers–cement pastes in concrete limits the scattering and motion of paste of cement and increase the viscosity of blends. With the increase in fiber content, the ability of interfacial bond between the fiber–cement pastes get resilient as extra fibers consume the paste of cement to cover over it. Therefore, the concrete workability reduced as the percentage of fiber expanded from 0% to 8%. Mehta and Monteiro [42] announced lightweight aggregate concrete with slump value between 50–75 mm is like conventional concrete with a 100–125 mm slump value. On account of PSC, the value of slump of glass fiber- and nylon fiber-reinforced PSC somewhere in the range of 60 mm and 65 mm can be compacted.

Figure 3 additionally displays that two kinds of fibers that affect the workability of concrete. The glass fiber created higher values of slump marginally than the nylon fiber. An increment in slump values at a scope of 5–10 mm and the highest variance among the glass fiber and nylon fiber were seen at 6% of fibers, and the value of slump of G6 is 9% more as compared to N6. Song et al. [43] studied that the fiber’s workability with a more limited length was less as compared to longer fibers. The surface area of nylon fibers is more to reinforce the bond between fiber and cement paste [15]. As the glass fiber length is more as compared to nylon fiber and, glass fiber has a lesser effective surface area to build up a bond of fiber cement as in comparison to nylon fiber. Slump flow of glass fiber reinforced concrete were somewhat higher than that of nylon fiber. Additionally, glass fiber is less ductile (more rigid), which offers more resistance to flow of concrete, which results in less workable concrete [22].

### 4.2. Density (Unit Weight)

**Figure 4:**
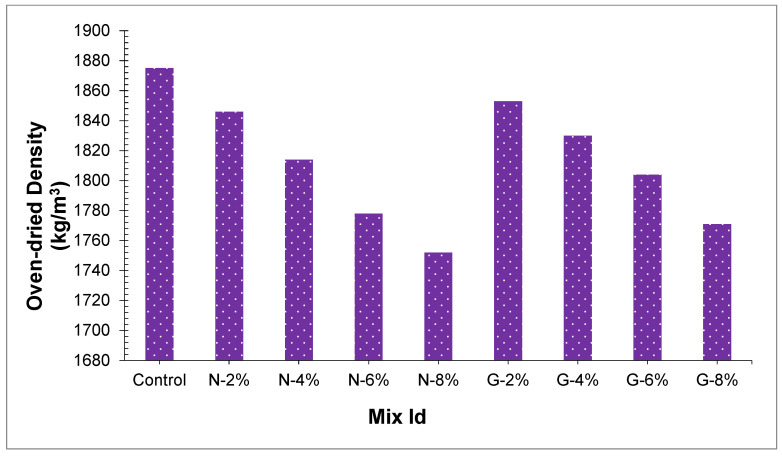
Oven-dried density of all concrete samples.

Lightweight aggregate concrete (LWAC) is described as concrete that has an oven-dried density in the scope of 1700–200 kg/m^3^ and more than 15 MPa of compressive strength [44]. The connection between the amount of fiber and oven-dried density has appeared in Figure 4. Outcomes appeared that the entire PSC oven-dried density went from 1801 kg/m^3^ to 1914 kg/m^3^, which satisfied the (LWAC) necessity because the peach shell is about 67% lighter than the normal weight aggregate. The inclusion of nylon and glass fibers in concrete decreased the oven-dried density of PSC because the specific gravity of fibers is low. When the percentage of nylon fibers is included at rates of 2%, 4%, 6%, and 8% by weight, concrete oven-dried density is reduced by 1.6%, 3.5% 5.3%, and 6.6%, separately, as in comparison to reference sample.

Figure 4 likewise indicated that the various sorts and volume parts of fibers impacted the peach shell concrete oven-dried density. It is concluded that concrete with glass fibers created a somewhat higher oven-dried density than nylon fibers, and the distinction in the peach shell concrete oven-dried density is more critical as the fiber percentage part goes from 2% to 8%. At 8% of glass fibers, G8 had the most minimal oven-dried density of 1771 kg/m^3^, and it was just 1.2% more than N8. It is noticed that though nylon fibers and glass fiber’s specific gravity is fundamentally diverse, its effect on the PSC oven-dried density is not self-evident. Nonetheless, compared with a unit weight of around 2410 kg/m^3^ for conventional concrete, PSC oven-dried density is reduced around 31%, which is a huge decrease in the weight of concrete.

### 4.3. Compressive Strength

**Figure 5:**
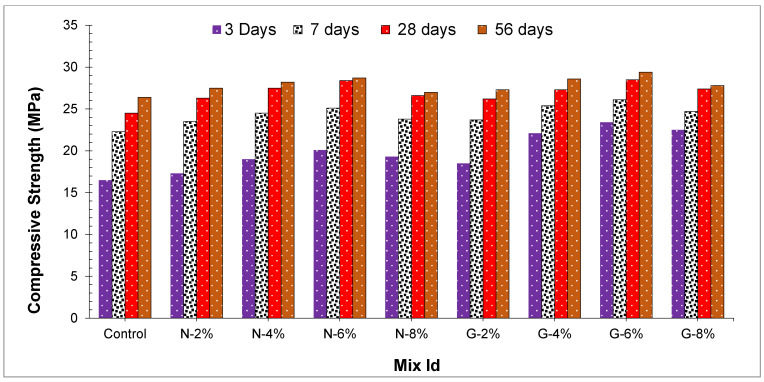
Compressive strength of concrete.

In Figure 5, the entire sample’s compressive strength is displayed. Experimental outcomes show compressive strength of concrete samples is improved as the time of curing of concrete is increased.

From Figure 5, it can be seen that there is a critical variation in the compressive strength amid peach shell concrete comprising various types and the ratio of fibers and reference mix with no fibers. The improvement of a definitive compressive strength might be recognized in the way that strands capture the development of the cracks because of the interfacial bond of fiber-cement paste and the crack bridging of fibers [30]. The expansion of nylon fibers and glass fibers had altogether improved the peach shell concrete compressive strength at all days of curing, the compressive strength improved as an expansion in fiber content. All blends at 28-days compressive strength differed between 17.3 MPa and 28.7 MPa that satisfied necessities for the density and strength of lightweight aggregate concrete. It may be seen that as in comparison with the reference blend, the compressive strength at 56 days of N2, N4, N6, and N8 expanded by 4%, 6.38%, 8.1%, and 2.3%, and compressive strength at 56 days of G2, G4, G6, and G8 expanded by 3.29%, 7.75%, and 10.20%, separately. At 6% glass fibers, the glass-fiber-reinforced PSC at 28-day compressive strength was marginally more than as compared to nylon fiber. This showed that utilization of glass fiber-reinforced peach shell concrete is a decent decision.

Figure 5 displays the compressive strength of the entire sample. At 3 days, the samples achieved around 61–77% of compressive strength, and at 7 days, the samples achieved about 84–89% of compressive strength. It showed that all peach shell concrete grew high at initial compressive strength because the consolidation of Silica Fume expanded the cement paste cohesiveness and decreased the micro-cracks advancing, and ultimately peach shell concrete early compressive strength is expanded. Expansion of nylon and glass fibers in concrete expanded the compressive strength of 56 days by about 5–9%. G6 led to the highest compressive strength of 29.4 MPa at 56 days of curing, which is 10.20% more as compared to the reference sample of 26.4 MPa. Although few out of every odd type of lightweight aggregate is appropriate for the making of lightweight aggregate concrete, the outcomes dependent on this investigation show that it is attainable to create lightweight aggregate concrete utilizing peach shell as a lightweight aggregate.

For the most part, the beginning and cracks spreading in concrete are because of the ceaseless expansion in compressive loading. Since the pressure produced by the fiber is opposite to the break spread way, the debonding starts at the interface among the cement paste and fiber. The fiber resembles an extension in this cycle that captures the propelling breaks and thus improves the concrete strength [45]. Therefore, the expansion of nylon and glass fibers improved the holding of the fiber cement paste interface and enhanced the concrete compressive strength [43]. Glass fiber-reinforced peach shell concrete compressive strength is more as compared to nylon fiber-reinforced peach shell concrete. This reason might be clarified as the elastic strength of glass fibers in this research is 2.5 times as compared to nylon fibers, the unit weight of glass fiber-reinforced PSC in this examination is higher than that of nylon fiber-reinforced peach shell concrete. Although the expansion of fibers in this research decreased the unit weight of peach shell concrete. For conventional concrete, the more the unit weight the more the compressive strength. Thus, higher unit weight of glass fiber-reinforced peach shell concrete compressive strength is more as compared to nylon fiber-reinforced peach shell concrete.

**Figure 6:**
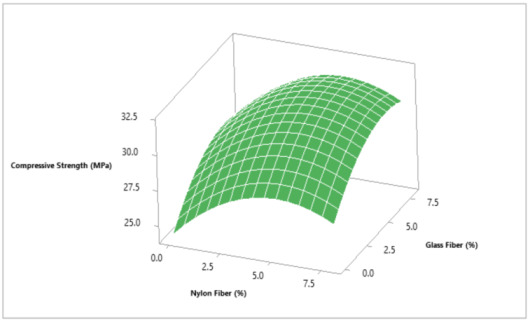
Three-dimensional response surface for compressive strength (MPa).

**Figure 7:**
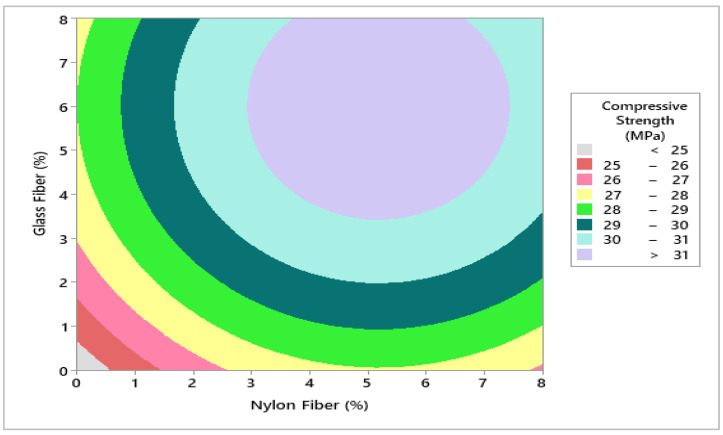
Contour plot for compressive strength (MPa).

Response surface methodology (RSM) is a statistical tool, and its main purpose is to predict response or output from the experimental trial tests, (RSM) can be influenced by several factors or input variables. When there is more than one response, then it is important to find the combined optimum dosage of both materials that is not possible individually [35]. In this research, Minitab software was used to develop a 3D response surface and contour plot to assess the combined effects of glass fibers and nylon fibers versus compressive strength. To evaluate optimum dosage for both fibers in concrete, 3D response surface was converted into contour plot in which 6.0% glass fibers and 5.5% nylon fibers were selected from contour plot giving highest compressive strength of 32 MPa as shown in Figure 6 and Figure 7. It also indicates that strength increases as the percentage of glass fibers and nylon fibers increase, giving a maximum strength of 32 MPa at 6.0% glass fibers and 5.5% nylon fibers. To validate the calculated value of statistical models for compressive strength of concrete, a similar dosage of 6.0% glass fibers and 5.5% nylon fibers batch were cast and experimentally tested in a laboratory for similar curing days. It was revealed that that the experimental value closely agreed with the predicted value, which validates the calculated response surface models.

### 4.4. Split Tensile and Flexural Strength

**Figure 8:**
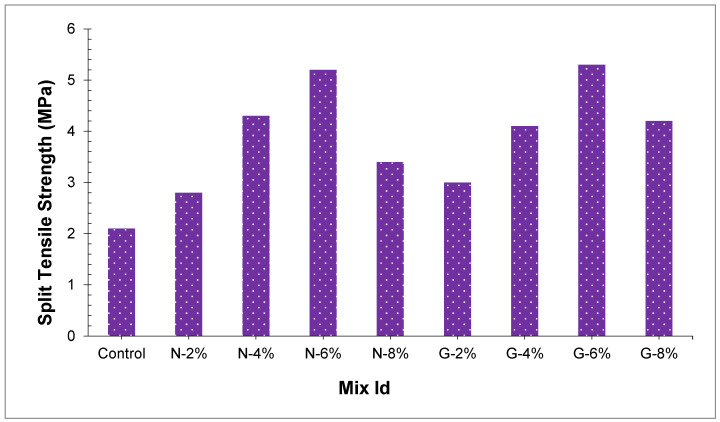
Split tensile strength of concrete.

**Figure 9:**
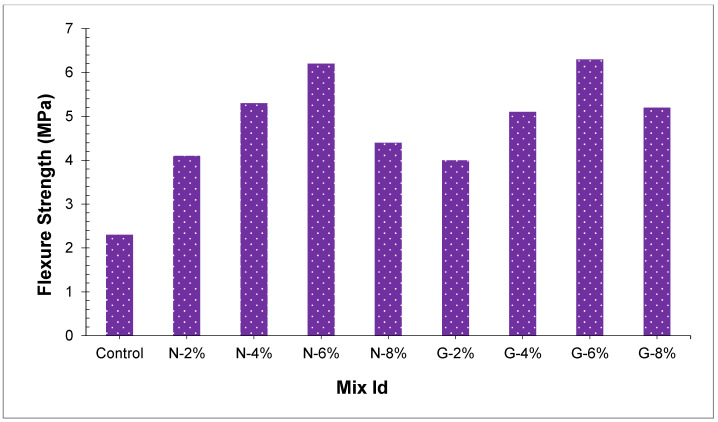
Flexural strength of concrete.

Entire concrete samples split tensile and flexural strength have appeared in Figure 8 and Figure 9, respectively. Cominoli [46] revealed that the inclusion of nylon fibers in concrete can somewhat improve the concrete flexural strength. Fiber enhances the concrete strength by the bridging effect and carrying part of stress [47]. Outcomes demonstrated that in concrete, the joining of glass fibers and nylon fibers enhanced both peach shell concrete split tensile and flexural strength, and the higher the amount of the fibers, the more the flexural and split tensile strength. Inclusion of nylon fibers from 2.0% to 8.0% and glass fibers from 2.0% to 8.0% enhanced the split tensile strength up to 9.8–57.2% and 8.0–60.37%. Moreover, glass and nylon fiber-reinforced concrete flexural strength was additionally improved. Peach shell concrete flexural strength enhanced as we increased the content of fibers. When the fiber percentages differ from 2.0% to 8.0%, the flexural strength for glass fiber and nylon fiber reinforced peach shell concrete enhanced by 7.1–63.49% and 6.2–39.4%, individually. The preferred position of peach shell concrete might be additionally clarified dependent on Figure 8. It was apparent from Figure 8 that the reference sample without any fibers failed and split tensile strength was reached to limit because of reduced ductility. Moreover, the fiber-reinforced peach shell concrete demonstrated phenomenal crack arresting performance even after the failing load sample is not broken into various parts.

Oven dry densities of N8 and G8 were lesser in the present study, and the flexural strength and split tensile strength of N8 and G8 were more as compared to the control sample. The ductile behavior of glass fibers is more prominent as compared to nylon fibers, glass fibers reinforced peach shell concrete led to higher flexural strength and split tensile strength. At percentages of 6% and 8%, flexural strength and split tensile strength of peach shell concrete that contain glass fibers were discovered to be about 8.1–16.8%, and 12.0–12.5% individually, and higher than those of nylon fibers. Flexural strength and split tensile strength of G6 were 6.3 MPa and 5.3 MPa, which improved by 63.49% and 60.37%, as compared with the control sample. At the same fiber percentage, the diameter of glass fiber is smaller, prompting more fibers to join the breaks [47]. Due to the fibers’ bridging effect, the development of cracks is hindered and around cracks, stress concentration is decreased. In the end, peach shell concrete tensile strength is improved.

**Figure 10:**
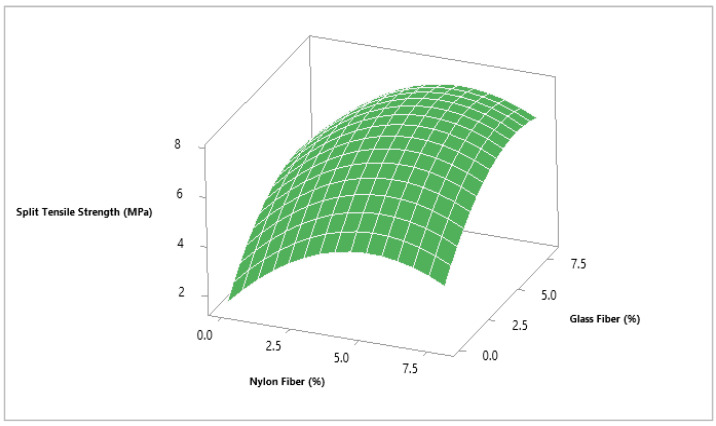
Three-dimensional response surface for flexural strength.

**Figure 11:**
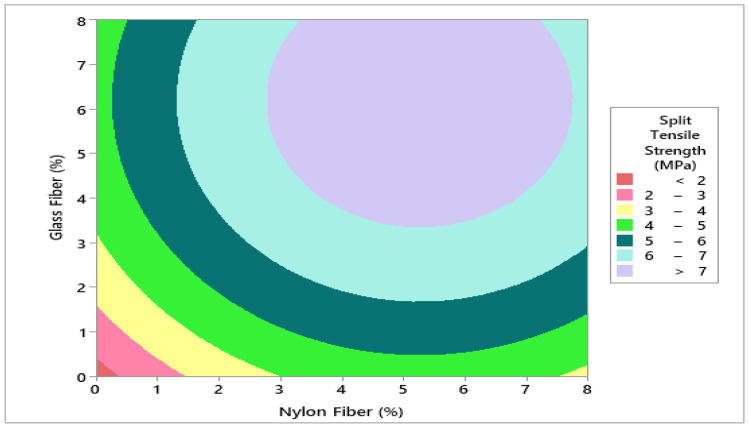
Contour plot for split tensile strength.

To evaluate the optimum dosage of glass fibers and nylon fiber for concrete split tensile strength, 6.0% glass fibers and 5.5% nylon fibers were selected from the contour plot giving split tensile strength 7.0 MPa as shown in Figure 10 and Figure 11. To validate the calculated value of statistical models for split tensile strength of concrete, a similar dosage of 6.0% glass fibers and 6% nylon fibers were cast and experimentally tested in the laboratory on the same days of curing. It was revealed that that the experimental value closely agreed with the predicted value, which validates the calculated values from response surface models.

**Figure 12:**
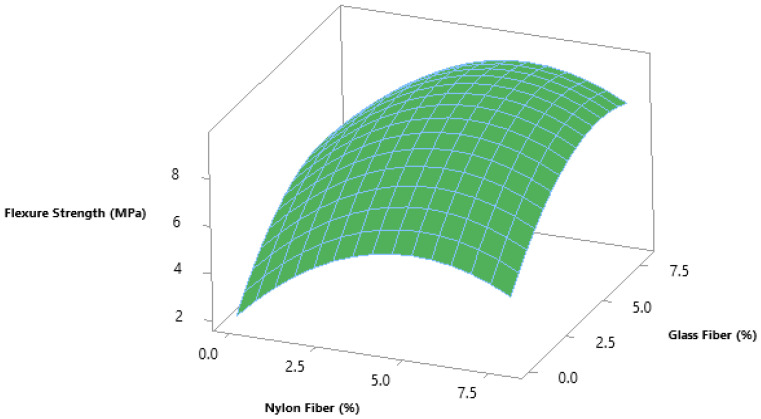
Three-dimensional response surface for flexural strength.

**Figure 13:**
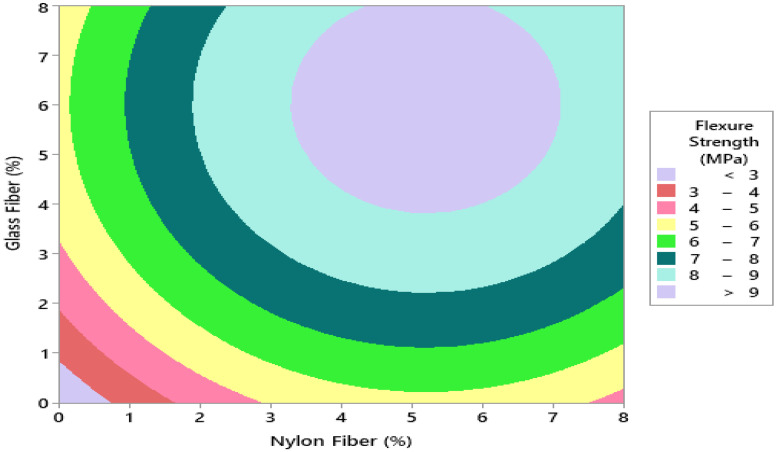
Contour plot for flexural strength.

To assess optimum dosage glass fibers and nylon fiber for flexure strength of concrete, 6.0% glass fibers and 5.5% nylon fibers were selected from the contour plot giving flexure strength 10 MPa, as shown in Figure 12 and Figure 13. To validate the calculated value of statistical models for flexure strength of concrete, a similar dosage of 6.0% glass fibers and 6% nylon fibers were cast and experimentally tested in the laboratory at the same curing days. It was observed that that the experimental values closely agreed with the predicted value, which confirms the calculated values from response surface models.

### 4.5. Modulus of Elasticity

**Figure 14:**
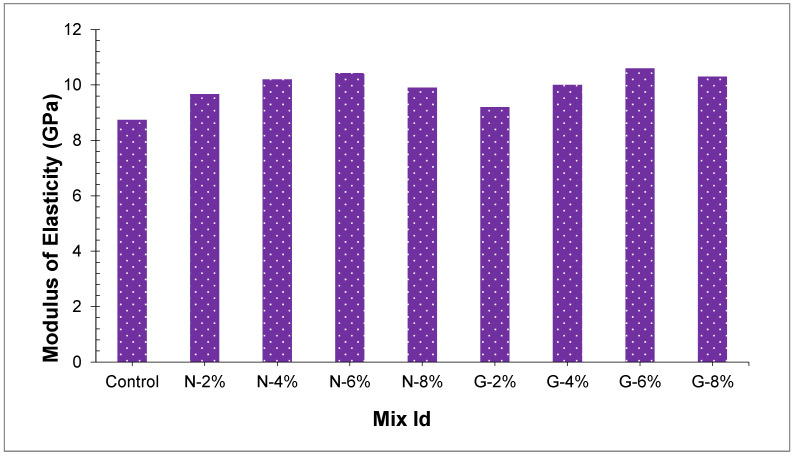
Modulus of elasticity of all concrete samples.

The modulus of elasticity (MOE) of all concrete samples is displayed in Figure 14, which ranges somewhere in the scope of 8.74 and 10.6 GPa. The reference sample in this examination delivered a base modulus of elasticity of 8.74 GPa. The outcomes showed that the glass fibers and nylon fibers significantly affected the modulus of elasticity. At percentages of 2.0%, 4.0%, 6.0%, and 8.0%, nylon and glass fiber-reinforced PSC modulus of elasticity expanded by 11.2%, 13.1%, 14.6%, and 15.4% for nylon fibers and 22.6%, 23.9%, 26.5%, and 29.8% for glass fibers. Adding of fibers enhances peach shell concrete modulus of elasticity since fibers capture the initial cracks brought about by shrinkage, and the strain is reduced because of the crack bridging effect brought by loading of compression and thus improves the modulus of elasticity. Modulus of elasticity of peach shell fiber reinforced concrete was found to rely upon the fiber content, while the kind of fibers impacted it. The content of glass fibers increased from 2.0% to 8.0%, the modulus of elasticity of glass fiber reinforced peach shell concrete expanded by 16.4% from 8.8 GPa to 10.6 GPa, and the modulus of elasticity of glass fiber reinforced peach shell concrete was 2.1%, 3.4%, 6.1%, and 4.7%, marginally higher than that compressive strength of nylon fiber PS fiber-reinforced concrete, and the modulus of elasticity of nylon fiber-reinforced PSC and glass fiber-reinforced peach shell concrete might be anticipated by compressive strength at 28-day.

### 4.6. Residual Compressive Strength

**Figure 15:**
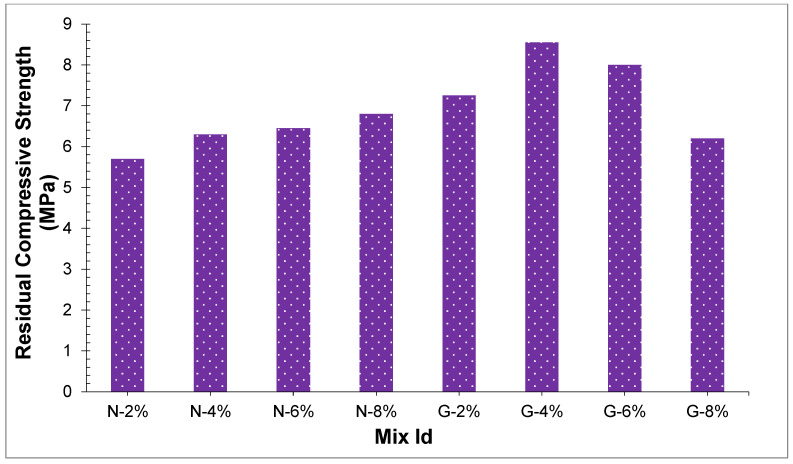
Residual compressive strength of concrete samples.

Figure 15 displays the residual compressive strength of the concrete sample. At two crack surfaces, the crack bridging effect of fibers existed, while in the fiber reinforced concrete, extra forces are required for additional development of the breaks or cracks. The joining of nylon and glass fibers improved the residual strength of PSC. Reference sample with no strands had no residual compressive strength when an ultimate loading strength is reached in the reference sample, it failed right away, while with the additional the fiber amount, the higher the value of residual compressive strength. At 6%, and glass fiber-reinforced PSC had the higher value of residual compressive strength, and nylon fibers had the highest compressive strength at 8%. It very well may be ascribed that the fibers’ crack bridging effect exists at two crack surfaces, which can block more cracks dispersion. Results demonstrated that the impact of including fibers is the reason for the enhancement of peach shell concrete post-failure toughness.

### 4.7. Porosity and Water Absorption

**Figure 16:**
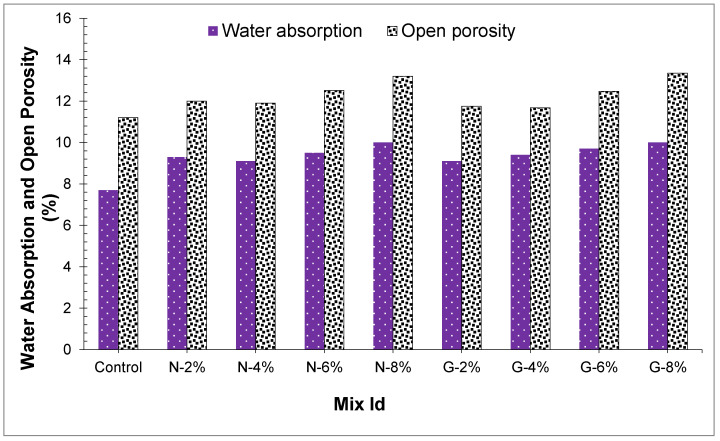
Water absorption and open porosity of concrete samples.

Water absorption and porosity for all blends of 24 h have appeared in Figure 16. Lo et al. [48] revealed that lightweight aggregate water absorption influenced the interfacial zone of concrete and hardened mortar internal microstructure, and a rise in lightweight aggregate water absorption brought an increase in pores amount in the interfacial zone of concrete. Figure 16 displays that the joining of fibers expanded in the peach shell concrete open porosity and water absorption, and all concrete sample’s water absorption values shifted from 8.1% to 9.7%. Fibers had a negligible impact on open porosity and water absorption; furthermore, with a higher the number of open pores, then all concrete samples’ water absorption will be more. Neville and Brooks [49] revealed that water absorption cannot be utilized to decide the concrete nature, the majority of the great quality concretes water absorption normally is less than 12% by mass. From Figure 16, it is shown that water absorption of all concretes has under 12% water absorption.

**Figure 17:**
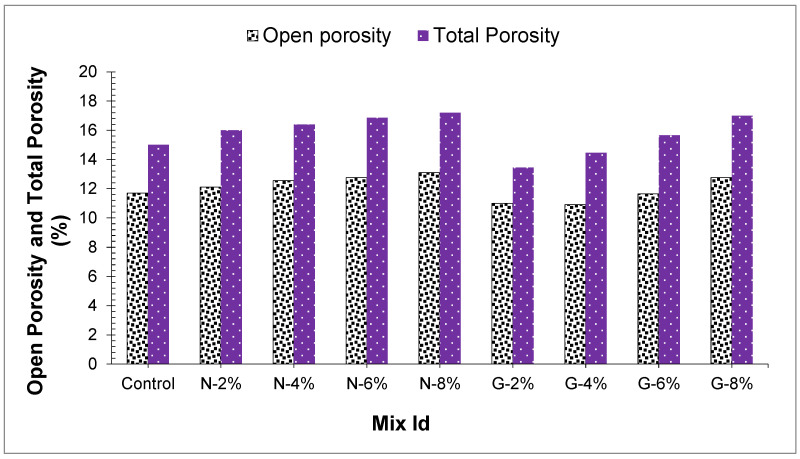
Open porosity and Total porosity of concrete samples.

In Figure 17, concretes’ open and total porosity have appeared. The outcome revealed that peach shell concrete total porosity was somewhere in the range of 14.8% and 17.1%, and the open porosity shifted from 11.1% to 12.9%. Moreover, the greater part of the peach shell concrete total porosity was open porosity, and closed porosity represented a little part of the total porosity because the outside of peach shell aggregate comprises a great deal of connective and micropore structures. Outcomes displayed that fibers had an inconsequential impact on the porosity of PSC, and the expansion of fibers marginally expanded the peach shell concrete porosity.

## 5. Conclusions

Effects of joining two different kinds and percentages of fiber (2.0%, 4.0%, 6.0%, and 8.0%) on the peach shell lightweight concrete mechanical properties have been studied in the present research. Following conclusions are obtained from the current study:

- Nylon and glass fibers reduce the PSC slump values. Fresh Peach shell concrete with nylon and glass fibers slump values are decreased to 60–70 mm. Concrete that has glass fibers had higher slump values as compared to nylon fibers.
- The oven-dried density of peach shell concrete is reduced by adding nylon and glass fibers. Concrete oven-dried density ranges from 1801 kg/m^3^ to 1914 kg/m^3^ and satisfies the lightweight aggregate concrete requirement.
- The early compressive strength of whole concrete samples was high. PSC compressive strength was increased with nylon fibers and glass fibers, and peach shell concrete compressive strength increments as we increase the fiber amount. Peach shell concrete compressive strength at 28 days was around 7–20%.
- Peach shell concrete split tensile strength is increased with adding of nylon fibers and glass fibers. Split tensile strength gets higher as the number of fibers is increased. Split tensile strength is increased by 9.8–57.2% and 8.0–60.3% by adding nylon and glass fibers from 2.0% to 8.0%.
- Peach shell concrete flexural strength improved as fiber content is increased. Flexural strength is increased by 6.2–39.4% and 7.1–63.4% by adding nylon and glass fibers from 2.0% to 8.0%.
- The flexural and split tensile strength of glass fiber-reinforced peach shell concrete is high. At the percentage of 6.0% and 8.0%, peach shell concrete containing glass fibers the split tensile and flexural strength are between 63.49% and 60.37% more as compared to nylon fibers.
- Statistical models (response surface methodology) confirmed the lab results, that glass and nylon could be utilized to improve the mechanical performance of peach shell concrete.
- Peach shell concrete modulus of elasticity is also improved with the addition of nylon fibers and glass fibers. All concrete samples’ modulus of elasticity ranges somewhere in the range of 10.2 GPa to 12.5 GPa. At percentages of 2.0%, 4.0%, 6.0%, and 8.0%, nylon and glass fiber-reinforced PSC modulus of elasticity enhanced by 11.2%, 13.1%, 14.6%, and 15.4 for nylon fibers and 22.6%, 23.9%, 26.5%, and 29.8% for glass fibers.
- No residual compressive strength was produced in a control sample. PSC residual compressive strength is increased by adding nylon and glass fibers. Furthermore, the higher the fiber amount, the higher the residual compressive strength. At a percentage of 8.0%, the residual compressive strength of nylon and glass fiber-reinforced PSC increases by 47% and 79%.
- Adding fibers marginally builds peach shell concrete porosity and water absorption. Despite that, the amount and type of fibers insignificantly affect water absorption and porosity.

The final conclusion about results obtained from the utilization of glass and nylon fibers is that both the fibers are suitable material to be used in concrete; however, in the present study, glass fibers were shown to be more effective in improving concrete mechanical properties.

## Figures and Tables

**Table 1:** Physical and chemical properties of Portland cement.

Chemical Composition	Percentage (%)	Physical Properties	Results
CaO	64.7	Size	≤75 microns
SiO_2_	23.9	Fineness	92%
Al_2_O_3_	5.4	Normal Consistency	31%
Fe_2_O_3_	3.7	Initial Setting Time	33 min
MgO	3.5	Final Setting Time	410 min
SO_3_	2.9	Specific surface	322 m^2^/kg
K_2_O	2.4	Soundness	1.70%
Na_2_O	1.2	28-days compressive Strength	42 MPa

**Table 2:** Physical properties of peach shell, coarse and fine aggregates.

Physical Property	Coarse Aggregate		Fine Aggregate
	Peach Shell (PS)	Natural Weight Aggregate (NWA)	Sand
Fineness Modulus	5	4.8	2.7
Specific gravity (g/cm^3^)	1.25	2.67	2.64
Water absorption (%)	27.64	0.7	1.4
Bulk density (kg/m^3^)	544	1552	1542
LA abrasion value (%)	5	26	-
Aggregate Impact Value (%)	1.89	17.21	-
Elongation Index (%)	62	33	-
Flakiness Index (%)	61	33	-

**Table 3:** Properties of glass and nylon.

Properties	Glass Fibers	Nylon Fibers
Color	White	White
Length (mm)	23	20
Diameter (um)	28	24
Aspect ratio	821	833
Density (g/cm^3^)	2.32	1.21
Tensile Strength (MPa)	1200	921
Elastic Modulus (GPa)	7.0	5.4

**Table 4:** Mix proportion of concrete.

Mix Code	Cement (kg/m^3^)	Silica Fume (kg/m^3^)	Sand (kg/m^3^)	Water (kg/m^3^)	SP (kg/m^3^)	PS (kg/m^3^)	Nylon Fiber (%)	Glass Fiber (%)
Contol	560	56	770	170	5.6	350	-	-
N-2%	560	56	770	170	5.6	350	2	-
N-4%	560	56	770	170	5.6	350	4	-
N-6%	560	56	770	170	5.6	350	6	-
N-8%	560	56	770	170	5.6	350	8	-
G-2%	560	56	770	170	5.6	350	-	2
G-4%	560	56	770	170	5.6	350	-	4
G-6%	560	56	770	170	5.6	350	-	6
G-8%	560	56	770	170	5.6	350	-	8

## Data Availability

The data required to support the present findings are present in the manuscript.
